# Can we estimate which colors our participants see? Comparing results from different gamma correction methods

**DOI:** 10.1177/20416695241278562

**Published:** 2024-09-15

**Authors:** Déborah Epicoco, Domicele Jonauskaite, Christine Mohr, C. Alejandro Parraga

**Affiliations:** Institute of Psychology, 27213 University of Lausanne, Lausanne, Switzerland; Institute of Psychology, 27213 University of Lausanne, Lausanne, Switzerland; Faculty of Psychology, 27258 University of Vienna, Vienna, Austria; Institute of Psychology, 27213 University of Lausanne, Lausanne, Switzerland; 518689 Computer Vision Center, Computer Science Department, 16719 Universitat Autònoma de Barcelona, Barcelona, Spain

**Keywords:** gamma correction, monitor calibration, online studies, methods in psychology, color studies

## Abstract

In experimental color research, one must ensure that color is displayed and described reliably. When monitors are involved, colors are displayed through device-dependent color systems. However, these values must be translated into device-independent color systems to interpret what people perceive, often involving techniques such as gamma correction. We sought to explore the feasibility of estimating gamma instead of relying on direct gamma measurements, which typically require specialized equipment like a chromameter. Potential solutions include a computerized perception-based gamma estimation task or adopting the industry-standard gamma value of 2.2. We compared these two solutions against the chromameter measurements in the context of a color-matching task. Thirty-nine participants visually matched red, yellow, green, and blue physical objects using a computerized color picker. Starting from these color choices, we applied two RGB-to-CIE*Lab* color conversion methods: one using a perception-based gamma estimation and another using the industry-standard gamma. Color values obtained with the chromameter differed from the other two methods by 6–15 JNDs. Small differences existed between the results obtained using the perception-based task and the industry-standard gamma. Thus, we conclude that when standard viewing conditions cannot be assumed, adopting a gamma value of 2.2 should suffice.

The public is interested in and receptive to advice on appropriate color choices as a wide number of popular marketing communications demonstrate. Websites offer services to either select the best colors for personal (Uzoezi, 2016) and communal (Medium, 2017) spaces or explain what colors mean (Kramer, 2022). Some even link colors to personality (Thomson, 2022) or mood (London Image Institute, 2020). The color and effect are also routinely associated in applied contexts, including therapeutic interventions (Iyendo et al., 2016; Lee et al., 2018; O’Connor, 2011; Rangel & Mont’Alvão, 2011). Yet, when trying to find out whether such popular claims are true, we encountered a scarcity of systematic empirical studies as well as heterogeneous findings (e.g., see Jonauskaite, Thalmayer, et al. (2021) testing popular color-personality links and Jonauskaite, Tremea, et al. (2020) testing a color therapy routine).

Studies that test the psychological and affective effects of color face many challenges due to variations in color and affect theories, as well as heterogeneous methodologies (for a discussion, see Elliot, 2015; Jonauskaite & Thorstenson, 2024; Mohr et al., 2018; Palmer Schloss, & Sammartino, 2013). A fundamental methodological challenge concerns color presentation itself. When studies are run in the laboratory, researchers can be precise in their color descriptions and presentations, whether shown physically or electronically on monitors (e.g., Brooker & Franklin, 2016; Jonauskaite, Camenzind, et al., 2021; Palmer et al., 2013; Tham et al., 2020; Wilms & Oberfeld, 2018; Witzel et al., 2019). They can control and report color parameters (e.g., hue, lightness/brightness/luminance, and saturation/chroma) and environmental parameters (e.g., lighting, reflections, viewing angle, etc.). However, when studies are performed outside well-equipped and well-financed laboratories, researchers face several obstacles, such as a lack of knowledge of the hardware and its precise setup, unpredictable changes in the environmental conditions, or being at a different location to participants (e.g., online studies). Such situations imply that researchers cannot control the exact stimuli their participants see. To experience first-hand what we mean, observe how the colors of a computer monitor change when viewed in the shade or sunlight, at different viewing angles, or under different ambient illumination conditions. Also, look at the same image on different monitors—despite the same orientation or exterior light conditions, the colors of these images can appear very different.

To explain such differences in color appearance, it is important to know that a color display can be unambiguously defined in *device-dependent* color systems, such as RGB, HSV, or HSL. However, an observer's final colors depend on several factors, such as the monitor, the monitor parameters, environmental conditions, and most importantly—its gamma curve (Parraga et al., 2014). Knowing the specifics of the gamma curve allows us to convert device-dependent colors to *device-independent* colors. Device-independent colors refer to a standardized color representation that is not influenced by the hardware used for display or reproduction. In practical terms, device-independent color spaces are defined based on perceptual attributes rather than specific device characteristics, making them more suitable for experiments with human participants, as is the case in psychology. Widely known examples of such device-independent color systems are the *Commission Internationale de l'éclairage* (CIE) *XYZ*, CIE*Lab*, CIE *Lch*, CIE *xyY*, and the Munsell Color System (Brainard et al., 2002; Carpenter & Robson, 1999).

The conversion between device-dependent and device-independent color systems may involve steps such as gamma correction, linear matrix mapping, or nonlinear transformations. To make a gamma correction, one must fit a power-law function (exponential response curve) to the luminance emitted by each of the red (R), green (G), and blue (B) components of each pixel of the monitor and do so as a function of an increasing pixel intensity value (Brainard et al., 2002; Carpenter et al., 1999). From this exponential response curve, we can compute the “decoding gamma,” or simply “gamma,” which ranges from 1.8–2.2 in modern computer displays (Parkin, 2018). This curve is a legacy of older cathode-ray tube (CRT) monitors, which convert electrical signals to light in a nonlinear way following the function of beam intensity vs. applied voltage in electron guns.

Most modern computer monitors assume the *industry-standard* gamma value of 2.2, which has been adopted by the Standard Red Green Blue system (sRGB), today's most popular device-dependent color system. The sRGB system was created by Hewlett Packard and Microsoft in 1996 for use in monitors, printers, and the Web (Poynton & Johnson, 2004). The standard value 2.2 is often considered the default, assuming a viewing environment that matches typical home and office viewing conditions (i.e., sRGB color standard and LCD monitors). In reality and in many cases, gamma values deviate from 2.2 since they are not strictly adopted by screen manufacturers, vary with off-axis viewing conditions, and their deviations become more pronounced over time (Parraga et al., 2014).

If one wishes to measure gamma rather than estimating it, there are at least two ways to determine the response curve of monitors: using a *light-sensitive device* (chromameter or spectrometer) or using a *perception-based task*. Light-sensitive devices measure the actual luminance of the R, G, and B pixel elements as a function of their grey-level intensity, allowing one to establish the exact response curve of a given monitor. While being the most accurate way to assess gamma, such instruments are expensive and require some training to handle. Without a chromameter, an alternative approach involves estimating the response curve through a perception-based task. In this specific task, trained observers visually compare two different areas of an image on the computer screen and adjust one of them by changing its luminance until the overall picture looks homogenous (see Colombo & Derrington, 2001, for an example of this procedure). Once gamma is established, the colors of interest can be converted from device-dependent to device-independent color systems.

Perception-based methods offer a cost-efficient approach to estimate the monitor's response curve, providing a potential alternative for screen color calibration. However, users need a level of training to effectively implement these methods, which requires controlled conditions for accurate execution. We tested their performance by contrasting the outcomes of two methods: (a) perception-based gamma estimation task and (b) applying the industry standard-based gamma correction against direct chromameter measurements.

We conducted a simple color-matching experiment in different environmental setups to replicate the naturally occurring noise in real-world situations. To make this exercise both feasible in time and plausible to be replicated in many places worldwide, we worked with widely available objects (Smarties sweets) that exist in distinct colors. Here, we chose the red, yellow, green, and blue Smarties. Importantly, all participants had to find monitor matches of the same colors of the Smarties (i.e., red, yellow, green, and blue), but some were in different viewing environments than others. To simulate different viewing conditions (i.e., under various levels of noise), we tested some participants under controlled laboratory conditions, others indoors at the local cafeteria, and others outside the university cafeteria. Some participants used their personal computers, while others used a computer from our laboratory.

Then, we converted participant-matched colors, defined in a device-dependent color system (i.e., RGB), to a device-independent color space (i.e., CIE*Lab*)^1^ using gamma corrections and algebraic transformations. We followed three different approaches to convert these colors: (1) chromameter measurements, (2) perception-based gamma estimation task, and (3) by using the industry standard 2.2 gamma value. Finally, we could determine how the values obtained with the perception-based and industry-based gamma measures differed from the color measures obtained with the chromameter. We expected the chromameter measures (which did not require a gamma correction) to be the most accurate. Hence, we adopted them as ground truth.

We worked under the assumption that the more time-consuming perception-based gamma estimation task would be worthwhile if the difference between its results and the chromameter measurements were smaller than the difference between those of the industry-standard task and those of the chromameter. It is important to note that our aim was not to assess the effectiveness of perception-based gamma correction or any specific screen calibration method under controlled conditions. Previous studies have already established their viability (see Colombo & Derrington, 2001). Instead, we focused on evaluating their practicability in real-world settings, where participants are responsible for calibrating their own screens and conducting experiments in uncontrolled environments (a common scenario in online experiments). We sought to determine the worth of undertaking such calibrations by gaining insights into these factors.

## Materials and Methods

### Participants

We recruited 40 first-year psychology students (six men) with a mean age of 19.8 years (*SD* = 1.6, range = 18–24). Participants had normal or corrected to normal vision and were not color-blind, as assessed with Ishihara's test for color deficiency (Ishihara, 1993). Participation was voluntary and remunerated with course credits. The study followed the principles expressed in the Declaration of Helsinki (Association, 2013), and no further ethics was required by cantonal law.

### Overview of the Experiment

**Figure 1.**
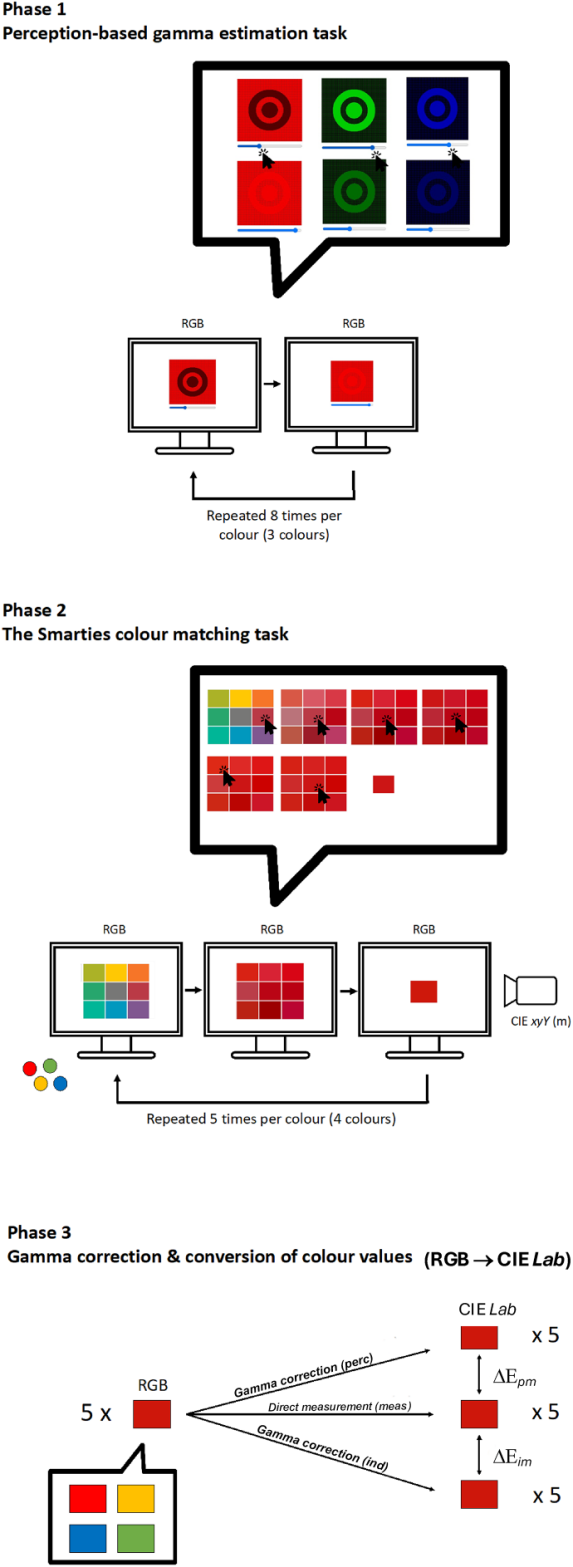
Schematics of our study. Phase 1: computerized perception-based gamma estimation task. This task was completed for the three screen color channels (red, green, and blue). Participants adjusted the given image so that the circle and the dot blended with the background. Each color channel was adjusted eight times, resulting in 24 adjustments in total. Phase 2: color matching task. Participants used a computerized color picker to match the colors of red, yellow, green, and blue Smartie sweet types. In the given example, participants took seven steps to match the color with the color picker. Each Smartie color was matched five times, resulting in 20 matched colors. After each match, the experimenter measured the matched screen color with the chromameter. Phase 3: gamma correction and color space transformation. We used the two gamma estimation methods: perception-based (*perc*), obtained in Phase 1, and industry-standard (*ind*), obtained with the standard gamma of 2.2, to convert the 20 matched colors in Phase 2 from RGB to CIE*Lab* color space.

Figure 1 provides the schematics of the experimental protocol. Participants had first to complete the perception-based gamma estimation task (Phase 1 in Figure 1). Then, they had to visually match the colors of the Smartie sweet types (Phase 2 in Figure 1) with a computerized color picker (Jonauskaite, Althaus, et al., 2019; Jonauskaite, Dael, et al., 2020; Jonauskaite et al., 2016). Once we obtained the device-dependent color values (RGB) from the color picker, we measured these screen choices with a chromameter (Phase 2 in Figure 1), obtaining the chromameter-measured values. For the other two gamma correction methods, we converted the RGB values obtained from the color picker into the device-independent color space—CIE*Lab* (Phase 3 in Figure 1).

### Phase 1: Perception-Based Gamma Estimation Task

**Figure A1.**
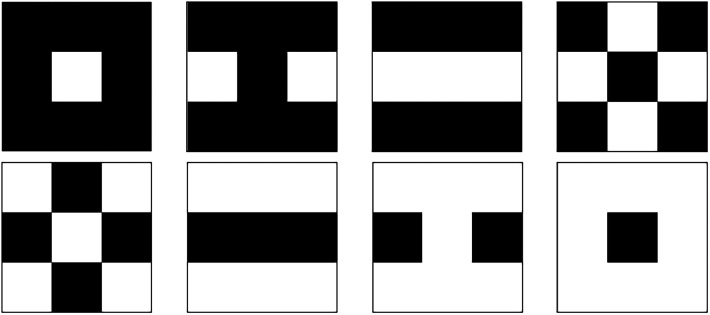
Eight half-tone patterns consisting each of 3 **× **3 pixels, emitting an increasing fraction of the maximum possible luminance. The fractions are 1/9, 2/9, 3/9, 4/9, 5/9, 6/9, 7/9, and 8/9 (from left to right, starting with the top row).

**Figure A2.**
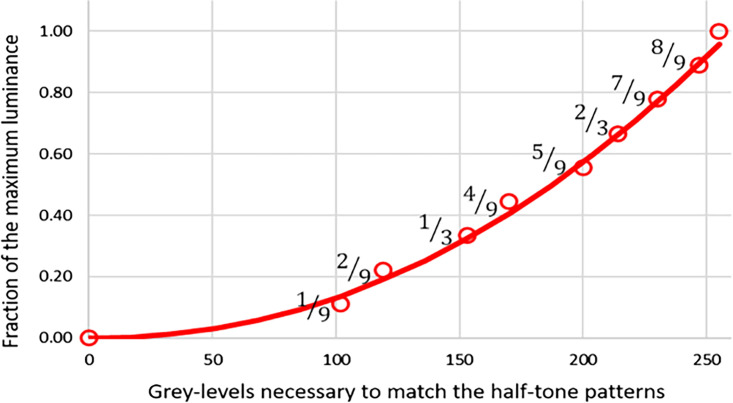
Simulation of a possible gamma curve obtained from fitting a power-law function to the results of a hypothetical participant performing the perception-based gamma estimation task.

Note. The small points represent the matching performance for each of the 3 × 3 pixel half-tone pattern, with an increasing fraction of the maximum luminance (see also Figure A1). The x-axis corresponds to the luminance value of the circle and ring, *y*-axis corresponds to the fraction of the maximal luminance of the monitor.

The preparation and implementation of Phase 1, the perception-based gamma estimation task, consisted of several steps adapted from Colombo and Derrington (2001). First, we created half-tone patterns that, when multiplied, resulted in the image material (see Appendix for details and Figure A1). Then, these images were implemented into the perception-based gamma estimation task (Figure 1, Phase 1).^2^ During this task, participants saw 24 square patches sequentially and in randomized order (see Phase 1 in Figure 1 and the Appendix for further explanation). They had to manipulate the circle and ring's brightness (i.e., perceived luminance) to match the background so that the entire patch appeared uniform in brightness (see Phase 1 in Figure 1). Participants could change the brightness of the circle and ring by moving the slider on the horizontal bar underneath the patch using the mouse or the keyboard (right and left arrow buttons). If the task was difficult to accomplish, we invited them to squint their eyes, which might help them perform it. When participants decided that the entire square patch appeared uniform in brightness, they continued to the next patch by clicking the “Next” button. For each participant and each square patch, we recorded the RGB values chosen for the circle and ring after being judged uniform (24 in total; see Phase 1 in Figure 1). Once participants completed the task (Figure 1, Phase 1), we used their individual results to estimate the gamma curve of their respective monitors, which was necessary to perform the color conversions later (Figure 1, Phase 3). Figure A2 shows a simulation of a possible gamma curve obtained from these results. The perceptual gamma estimation algorithm was originally implemented to accompany a color picker used in previous research (see below). At the time, it was tested against colorimetric measures in four different monitors (in controlled laboratory conditions), and its results were within a 5% tolerance limit.

### Phase 2: The Color Matching Task Using Smartie Sweet Types

#### Color Picker

This color picker is a user-friendly online program designed to facilitate the matching or selection of target colors (Jonauskaite et al., 2016; Jonauskaite, Althaus, et al., 2019; Jonauskaite, Dael, et al., 2019). With the color picker, participants can go through all the possible colors their computer monitor can produce.

The color picker starts with nine color patches, presented on a white background (see Phase 2 in Figure 1). Participants can select the color that most closely resembles a target color by clicking on the corresponding color patch. After the first color choice, participants can narrow the selection of color patches tailored to their first selection. The selected color appears in the middle, and eight patches of similar colors surround it. These eight patches vary along the axes of dark–light (lightness), red–green, and yellow–blue, and their properties are approximately based on the CIE*Lab* color system*.* This means that if a participant clicks on the upper left color patch, it will become yellower than before. The color choice can be fine-tuned by clicking on the outer corner patches (depicting variations in red-green and yellow–blue dimensions), the upper or lower middle patches (depicting higher or lower levels of lightness, respectively), or the right or left middle patches (depicting higher or lower levels of chroma, respectively). With each selection, the respective outer patch becomes the center patch on the next monitor. Participants can make as many selections as they need to arrive at their target color.

When they are satisfied with their choice, participants may click either on the central patch until it appears alone at the center of the monitor or, if they have made enough selections, the patch automatically appears alone at the center of the monitor. If participants want to modify their selection further, they can click again on the central patch to make the surrounding patches reappear and continue their selection. Once the target color is selected and confirmed, the responses are saved as RGB color values and the time and the number of clicks taken to reach the target color.

#### Color Matching Procedure

**Figure 2.**
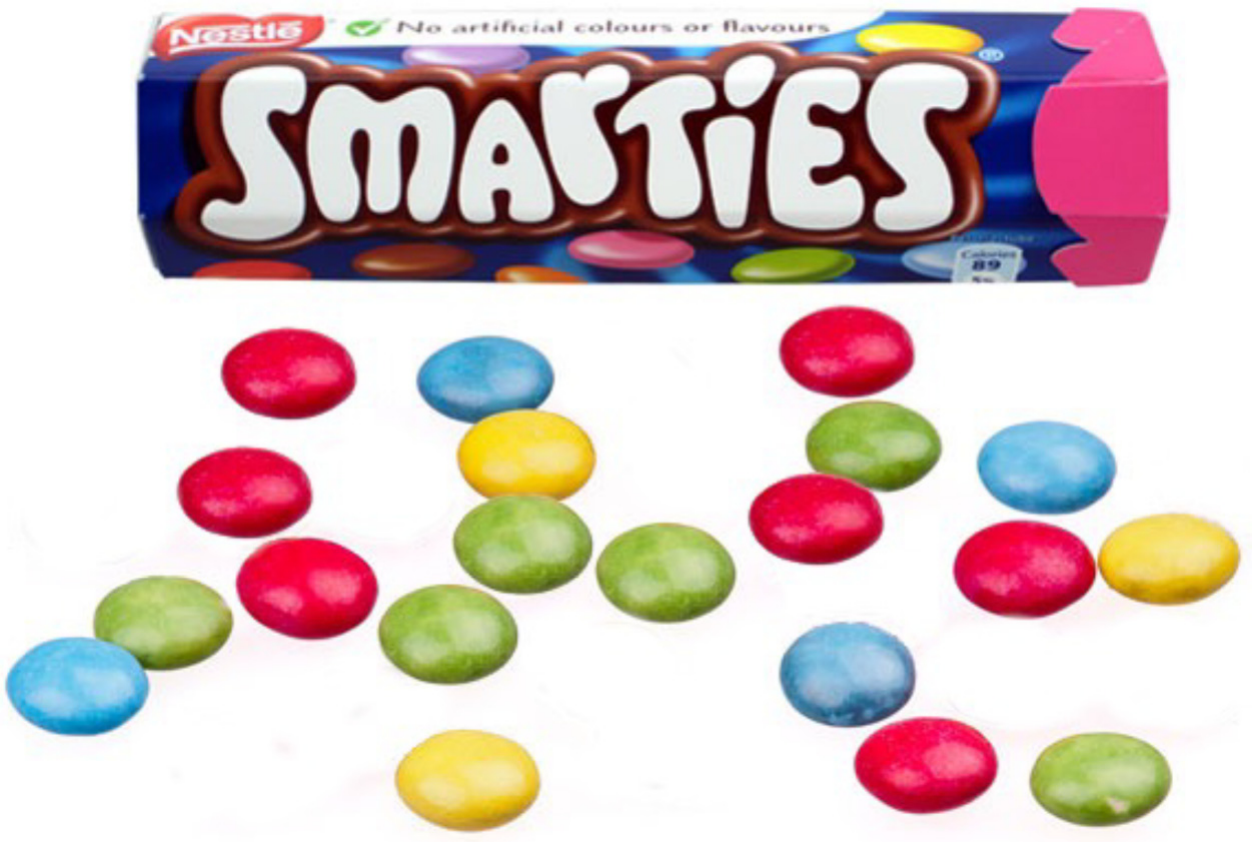
Examples of the Smartie sweet types (i.e., red, yellow, green, and blue) used in the experiment. The Smartie sweet types, manufactured by the Nestle company (https://www.nestle.com/brands/chocolate-confectionery/smarties), measure about 12 millimeters in diameter and 5 millimeters in thickness. Here, you can find the four colors we used in the study. We tested their color variability within the same hue and found it below 0.3 *CIELab* units, which is well below the standard JND.

We used four Smartie sweet types—red, yellow, green, and blue (Figure 2). We placed these four Smartie sweet types in front of the participants, to their left side (Phase 2 in Figure 1). Smarties measured approximately 12 mm in diameter and 5 mm in thickness. We chose Smartie sweet types as color-matching targets because (1) they are widely available and of relatively low cost, (2) their color variability was found to be below the Just Noticeable Difference (JND) of a typical human observer, and (3) their rounded shape produced specularities that also are present in many real-world objects. The commercial set of Smartie sweet types consists of eight different types (red, yellow, green, blue, orange, purple, pink, and brown). We selected red, yellow, green, and blue Smartie sweet types, which corresponded approximately to the hues of the screen's RGB channels and were relatively well spread in CIE*Lab*. Participants matched the color of each Smartie sweet type five times using the color picker. We randomized the order of presentation by shuffling 20 cards. Each card displayed one of the four color terms (red, yellow, green, or blue) and each presented colour term was repeated five times. Participants worked through the pile of cards at their own speed by taking the top card, reading its color term, looking at the respective Smartie sweet types without touching it, and matching its color with the color picker. After each color match, we measured the displayed color with a Konica Minolta CS-100A chromameter^3^, with an accuracy of 2% in luminance and ±0.004 in chromaticity. Then, participants took a new card from the top of the pile of cards and matched the next Smartie until the pile was finished. They saw all Smartie sweet types simultaneously, and there was no break between the selection of each Smartie sweet type.

### General Experimental Procedure

Participants were welcomed in the laboratory. They received written study information before signing the consent form. We then collected their demographic information and assessed color vision deficiencies with the Ishihara test (Ishihara, 1993). To test participants under varied environmental conditions, we allocated them randomly to either the “laboratory” or “outside laboratory” condition (between-subjects). In the laboratory condition, participants were accompanied to an individual dark testing room and were given a couple of minutes to adjust to the lighting condition before starting the experiment. Half of the participants used our computer (*n* = 10), while the other half used their own laptop (*n* = 10). In the outside the laboratory condition, we took participants to two different testing locations outside of the controlled laboratory conditions. Half the participants went to the local cafeteria (indoors) and sat at a random table (*n* = 10). The cafeteria had large windows and artificial ceiling illumination, resulting in varied light conditions depending on meteorological conditions and time of the day. The other half went to an outdoor location (the terrace of the local cafeteria) and again sat at a random table (*n* = 10). Of note, when testing outdoors, the lighting conditions naturally varied due to changes in weather and testing times. Thirteen participants used their own laptops, and seven used a laptop from the local laboratory—a 12-inch MacBook Retina (2017).

Irrespective of the testing condition (laboratory or outside of the laboratory), participants were seated in front of the computer monitor with a comfortable eye-monitor distance of around 30 cm. At the same time, this distance should ensure that participants see colors as similarly as possible over time. Consequently, they were asked to keep this distance constant throughout the experiment. The experimenter also ensured that participants’ eyes were at the same height as the central item on the monitor. Participants first completed the perception-based gamma estimation task and then performed the Smartie sweet types color-matching task (see Phases 1 and 2 in Figure 1). The entire experiment took around 30 min to complete, allowing to mitigate extensive natural variations as much as possible. At the end, participants were thanked and fully debriefed.

### Phase 3: Gamma Correction and Conversion of Color Values

**Figure 3.**
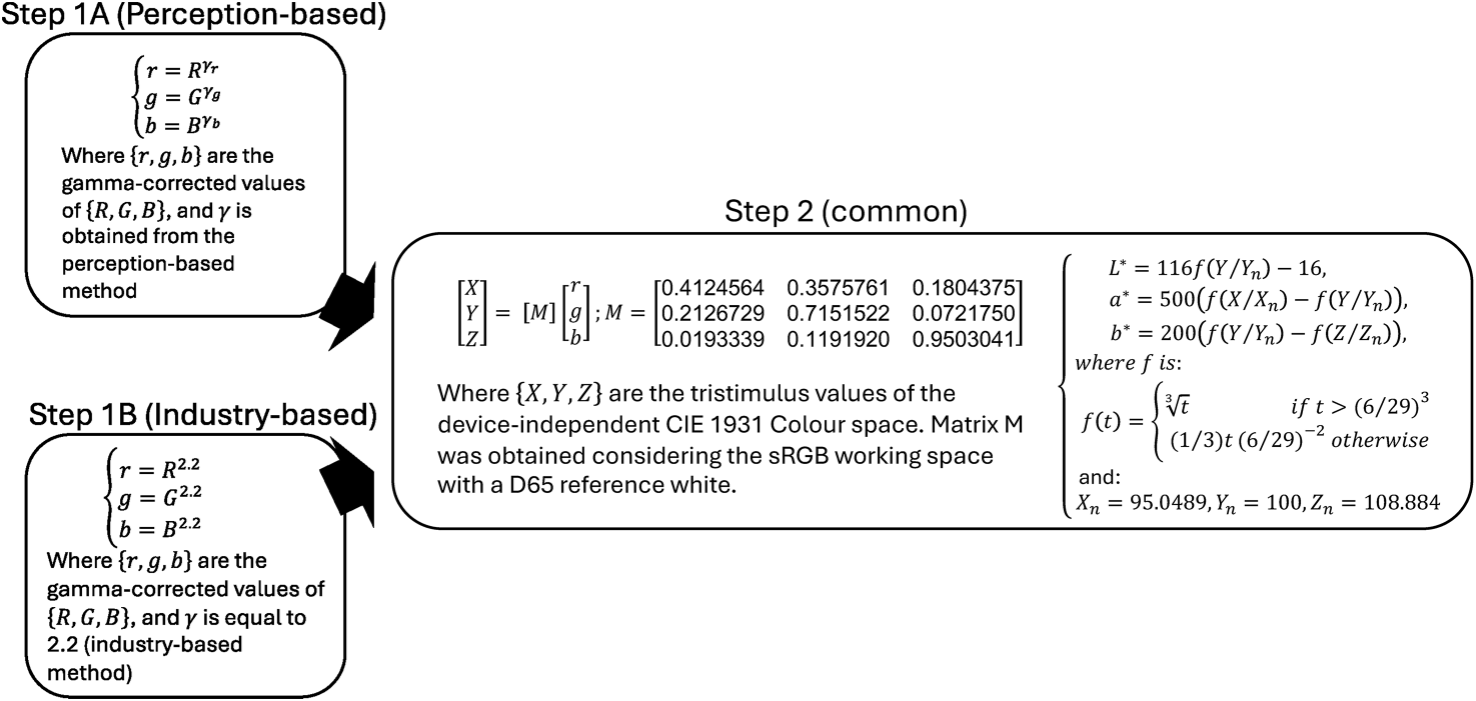
Representation of the gamma correction and the steps of the matrix color transformation. Step 1A, a perception-based gamma correction, includes the measurement of the three gamma values (γ_r_, γ_g_, γ_b_) corresponding to the three R, G, and B color channels. In contrast, in Step 1B, the industry-based gamma correction assumes a fixed value of 2.2. Step 2 consists of two parts: first, there is a matrix transformation to obtain colors in the CIE XYZ system, and second, there is a nonlinear algebraic transformation to convert them to the CIE*Lab* system. The figure shows the classical equations used in these transformations (Wyszecki & Stiles, 1982).

The key point in the current study is the comparison between the methods of color conversion from the device-dependent (RGB) to the device-independent (CIE*Lab*) color systems (see Phase 3 in Figure 1). Such color conversions involve two steps—gamma correction and color space transformation (Figure 3). Once colors are gamma-corrected, they can be converted from a device-dependent to a device-independent color system (usually CIE *XYZ*) using a matrix product. After that, they can be converted to any other device-independent color system, such as CIE*Lab* (which we use here), by applying a standard set of algebraic equations.

We made the following assumptions for the upcoming analyses to investigate the performance of monitor calibration methods outside the laboratory (i.e., presuming a very limited knowledge of the intervening hardware and environmental conditions). First, the chromaticity of the LCD monitor primaries is compatible with the standard defined in the sRGB color space (thus, the matrix transformation step is viable). Second, the screen settings are set to “default,” i.e., no custom chromaticity software is changing the color temperature of the screen according to the time of the day, the browser is set up to 100% pixel scaling, the maximum brightness is not far from 100 Cd/m^2^, etc. Third, the illumination is the CIE D65 standard daylight illuminant, which should be used in all colorimetric calculations requiring representative daylight (CIE, 1999) and was the Illuminant used in our laboratory.

We used three methods to convert target colors between the RGB and the CIE*Lab* color systems (see Phase 3 in Figure 1). The first method was the chromameter. After each color selection, we measured the picked screen colors using the chromameter and obtained their CIE *xyY* values (see the last step in Phase 2 in Figure 1). Then, we converted them to CIE*Lab* using the standard equations shown in Step 1B of Figure 3 (skipping the matrix multiplication). These were direct colorimetric measures, and we noted them as direct measurements (*meas*) in Phase 3 of Figure 1. The second method was the perception-based gamma estimation task shown as Gamma correction *(perc)* in Figure 1 and represented in Step 1A in Figure 3. The third method was the industry-standard gamma estimation task shown as Gamma correction *(ind)* in Figure 1 and represented in Step 1B of Figure 3. In the two latter cases, we assumed the sRGB model for our computer monitors and *CIE* Standard Illuminant D65 (average midday light) as a working reference white (Wyszecki & Stiles, 1982). For the perception-based gamma estimation task, we used the recorded RGB values for the circles and rings in Phase 1 to fit the power law function as the luminance intensity increased (Brainard et al., 2002; Carpenter et al., 1999). In this way, we derived the gamma values for each participant and each color channel—R, G, and B (see Phase 1 in Figure 1 and Appendix Figure A2 for an exemplary simulation of this process). For the industry-standard gamma, we assumed the value of 2.2. In both cases, we used gamma to convert the original RGB values to the gamma-corrected r, g, and b values (Steps 1A and B in Figure 3).

In the second step (Step 2 in Figure 3), we calculated a matrix product between the gamma-corrected r, g, and b values and a 3 × 3 transformation matrix that is standardized for these color models. In this way, we arrived at CIE *XYZ* color values. Finally, we arithmetically converted the CIE *XYZ* color values to CIE*Lab* using the rest of the formulae in Figure 3. The maximum luminance was obtained from the maximum value of “Y” (Luminance) measured on the screen by the chromameter in each session, which was close to 100 Cd/m^2^ and always corresponded to a yellow Smartie sweet type matching.

### Data Treatment and Analyses

We excluded three participants because part of their data got lost due to a technical glitch. Thus, the sections below include the complete sets of measures from 37 participants (six men). The Appendix presents the same results, excluding outlier measures from each group of five repetitions. The dataset analyzed during the current study is available here: https://osf.io/hs7p2/.

To test whether the time-consuming perception-based gamma estimation was worthwhile, we compared the colors obtained with (1) the perception-based gamma estimation vs. the chromameter and (2) the industry-standard vs. the chromameter. We analyzed these results regarding chromaticity differences, considering distances in the CIE*Lab* (*a,b*) plane and color differences (Δ*E*) considering all three dimensions of CIE*Lab*.

To test whether the means of the distributions were statistically different in the two-dimensional (*a,b*) chromaticity plane, we applied the Minimum Energy test (Aslan & Zech, 2005) and the T-Squared test (Mardia et al., 1979) for each Smartie sweet type separately. Thus, we could look at the distances between the distribution centers and their significance levels (*p*-value). If the three distributions of chromaticity values produced comparable results, the tests should not be significant, and the mean distances should be small (close to zero). However, if the three methods produced different results, then the tests would be significant (*p* < .050). Our approach was similar for the distribution of color values in the three dimensions of CIE*Lab*, except that we used the N-dimensional versions of the same significance tests.

Second, we calculated the difference between the colors obtained with the perception-based gamma estimation task and those obtained with the chromameter (Δ*E_pm_*), and the difference between the color values obtained with the industry-standard gamma and the same chromameter-measured colors (Δ*E_im_*). These calculations, which return a single positive number, are described below by Equations (1) and (2), respectively, and schematically illustrated in the Appendix, Figure A5.

(1) $$\Delta E_{\;pm}^{}=\sqrt{{\left(L_{p}-L_{m}\right)}^{2}+{\left(a_{p}-a_{m}\right)}^{2}+{\left(b_{p}-b_{m}\right)}^{2}}$$

(2) $$\Delta E_{im}^{}=\sqrt{{\left(L_{i}-L_{m}\right)}^{2}+{\left(a_{i}-a_{m}\right)}^{2}+{\left(b_{i}-b_{m}\right)}^{2}}$$

In an ideal world, both the perception-based gamma and the industry-standard gamma would yield color values identical to those measured by the chromameter, given the identical source color. In this perfect situation, the differences in Equations (1) and (2) would be zero. However, if they are larger than zero, then it is interesting to know which is the largest. Thus, we tested if the average $\Delta E_{\;pm}^{}$ values were statistically different from the average of $\Delta E_{im}^{}$ values using a paired-sample t-test.

## Results

**Figure 4.**
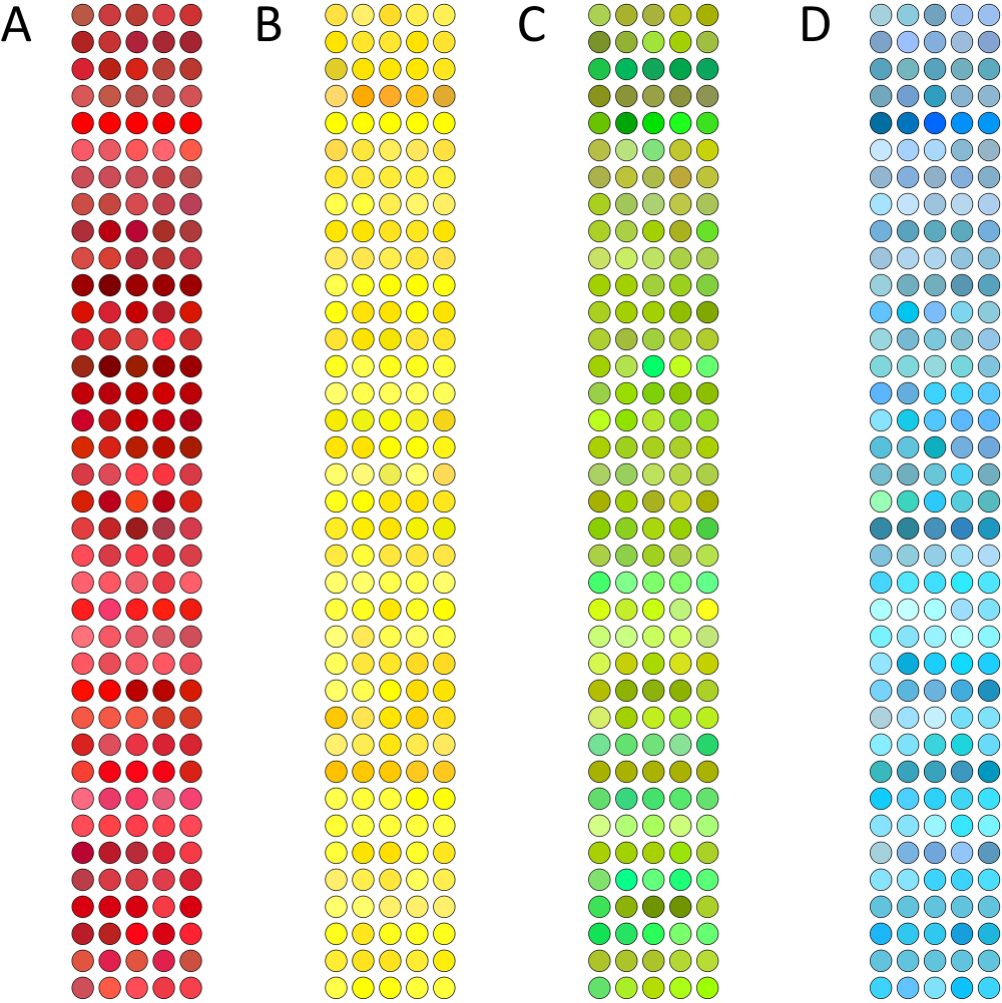
Representations of the color matches for red (A), yellow (B), green (C), and blue (D) Smartie sweet types. Each small circle symbolizes one color match, each row represents one participant, and each column represents one repetition (five repetitions for each Smartie sweet type). The figure colors are approximations to the actual colors obtained from each match.

**Figure 5.**
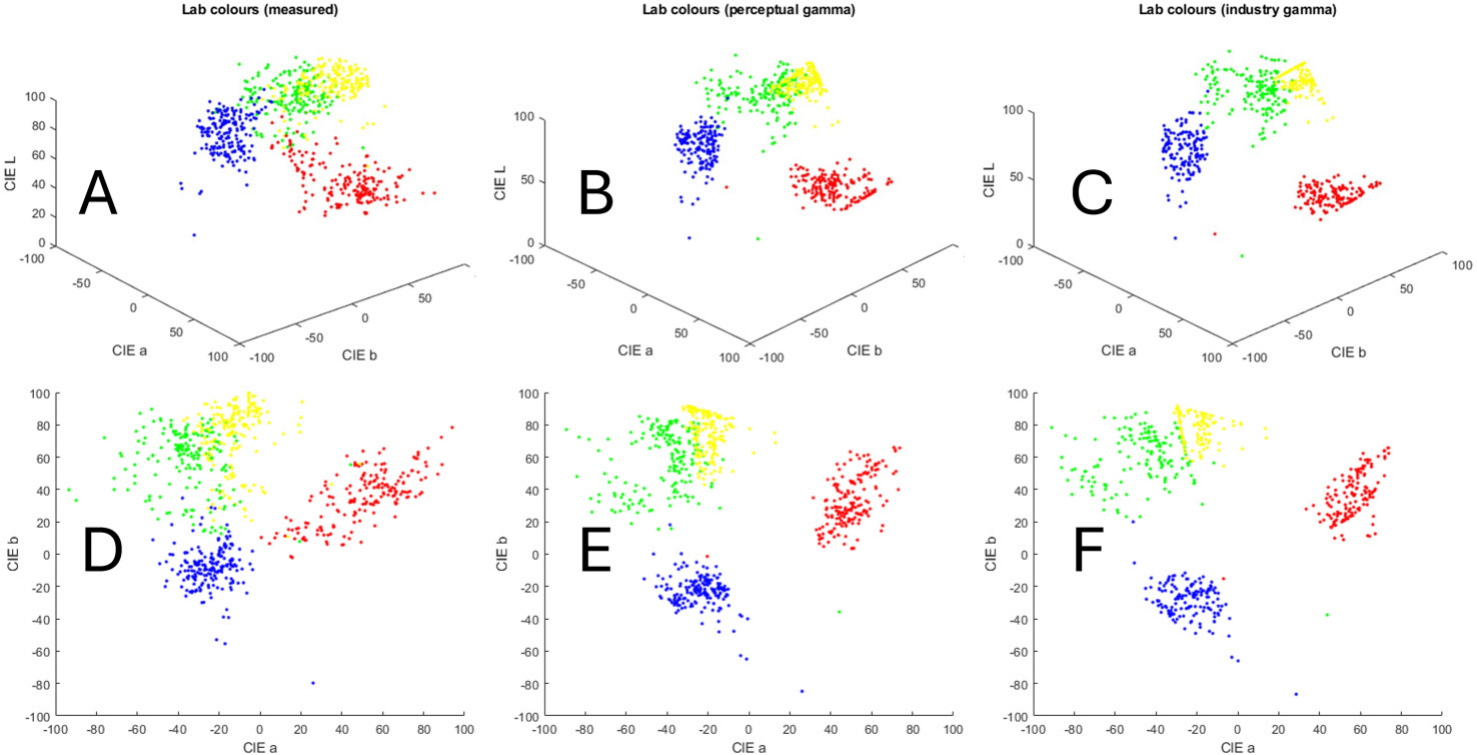
Distributions of color values for the matched blue, green, red, and yellow Smartie sweet types, separately for the three color-conversion methods (measured, perception, and industry). Panels A, B, and C represent the dots of Figure 4 in CIE*Lab* (three dimensions), and panels D, E and F represent their projections into the (a, b) plane of CIE*Lab* (chromaticity only).

Each of the 37 participants made five repetitions for each of the four Smartie sweet types, resulting in 20 color matches in total (see Figure 4). The observer selected a color (RGB values) for each match, and the experimenter measured it using the chromameter. We converted these RGB matches to CIE*Lab* using the two gamma estimation methods, following the schematics presented in Figure 3. After that, we converted the chromameter measures (originally in CIE *xyY*) to CIE*Lab*. Please note that the latest is a conversion between device-independent color spaces and does not require gamma correction or matrix multiplication. Figure 5 shows the same color matches and their corresponding chromameter measurements after their conversion into CIE*Lab*.

### Distribution of Chromaticity Values in the CIELab (a,b) Plane

**Figure 6.**
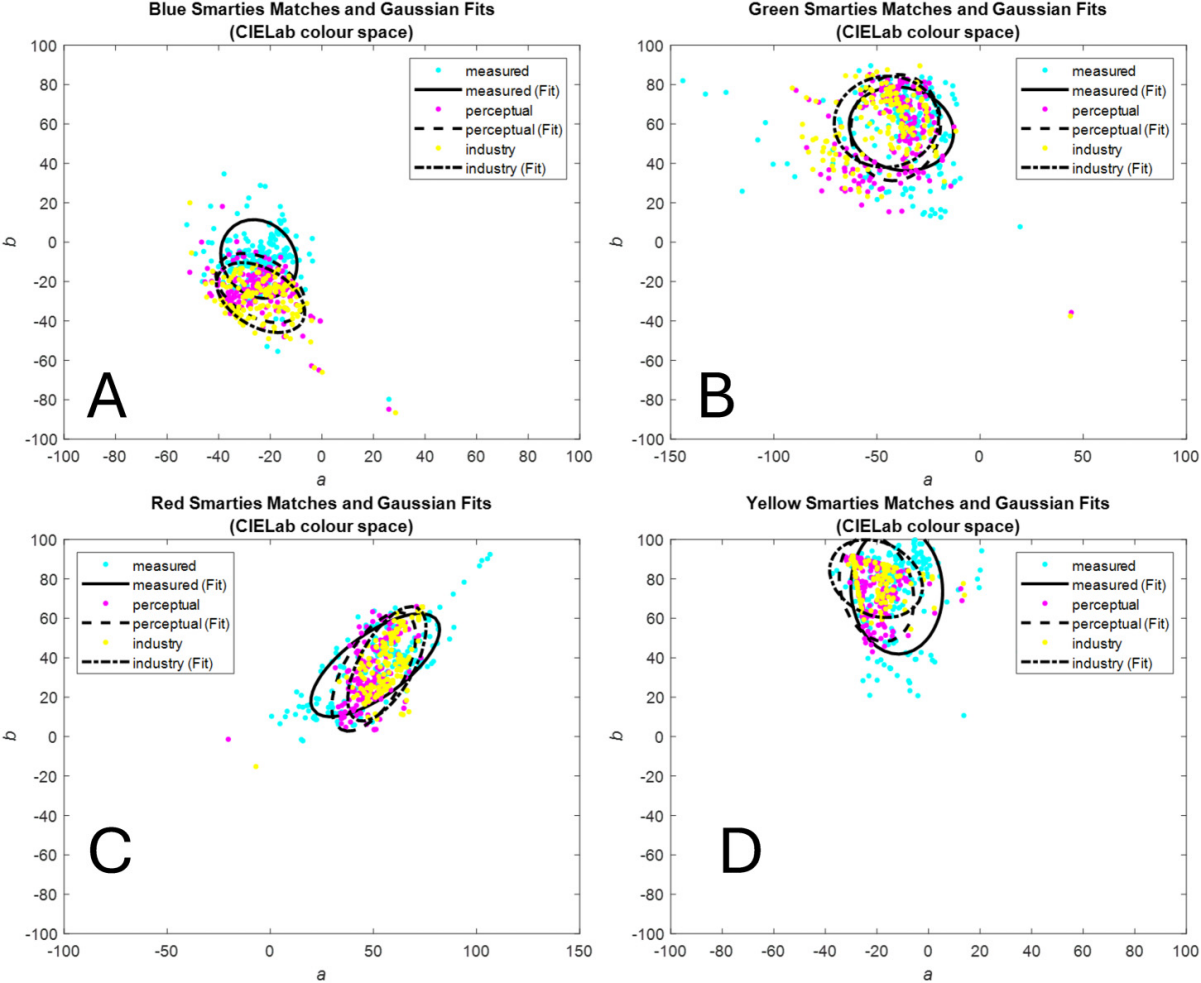
Multivariate Gaussian fits the matching results obtained for each of the different Smartie sweet types (blue, green, red, and yellow) in CIELab space. *Note*. The fits were obtained using the Matlab function “fitgmdist.m” based on the method proposed by McLachlan and Peel (2000). We used these distributions to run the Minimum energy and the Hotelling T-squared tests.

We compared the differences between the measured colors and those resulting from the gamma-conversion methods. Figure 6 shows the distributions of matched chromaticity values for the three methods (measured, perception, and industry) considering only CIE*Lab* (*a,b*) dimensions. Panels A, B, C, and D show the results for each of the four Smartie sweet types (blue, green, red, and yellow). In the same figure, we show the partially overlapping ellipsoids corresponding to their two-dimensional Gaussian distribution fits. Each distribution has an associated centroid, and we measured their pairwise distances. Table 1 shows the distances between the pairs of centroids in CIE*Lab* (*a,b*) and their significance for each Smartie sweet type according to the Minimum energy test and the Hotelling T-squared tests.

**Table 1.** The mean distance between the distributions of chromaticities in the CIE(*a*,*b*) plane, with their related *p*-values in CIE*Lab* units.

Smartie sweet type	Perception-based vs. measured	Industry-standard vs. measured
Blue	15.54*+	19.84*+
Green	2.90**+++	9.22*+
Red	3.66*++	5.30*+
Yellow	7.97*+	10.01*+

*Note.* The values represent the average distance between pairs of centroids in the chromatic plane of CIE*Lab*. Pairwise significance was calculated using the Minimum energy test (*p_m_*) and the Hotelling T-squared test (*p_t_*) for comparing two-dimensional data from two independent samples*.* Significance is coded as *p_m_ < .001, **p_m_ < .020, for the Minimum energy test and +*p_t_* < .001, ++ *p_t_* < .1 +++*p_t_* < .05 for the Hotelling T-squared test*. CIELab* is constructed so that a value of 2.3 units corresponds to the JND.

### Average Color Differences (ΔE) for all Measures, Considering all Three Dimensions of CIELab

**Figure A4.**
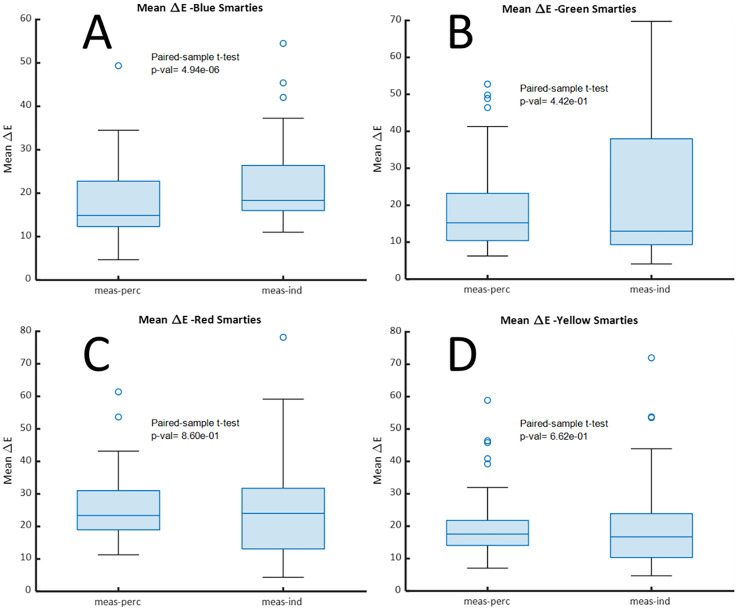
Variability of the color differences, by Smartie sweet type.

We applied Equations (1) and (2) to all the matchings and obtained the color difference for the pairs perception-measured and industry-measured. Since the color matchings were repeated five times, we obtained the means and standard deviation of the five color repetitions and applied our analysis to those. Table 2 shows the mean values of these color differences (*n* = 37 in all cases), and the boxplots in Figure A4 show the spread and central tendency of these color differences, discriminated by Smartie sweet types. We tested whether these values were statistically different (e.g., if the values in the first column differed from those in the second column), using the paired-sample *t*-test function provided by MATLAB. We also present a similar analysis in Table 3, considering only matchings made inside and outside the laboratory.

**Table 2.** Mean of all pairwise differences $\bar{\Delta E}$ (as defined by Equations (1) and (2), averaged over five repetitions), with their related *p*-values*.*

Smartie sweet type	Perception vs. Measured $\left(\bar{\Delta E_{\;pm}^{}}\right)$	Industry vs. Measured $\left(\bar{\Delta E_{im}^{}}\right)$	*p*
Blue	18.69	22.51	<.001
Green	20.33	21.65	.262
Red	26.25	26.38	.932
Yellow	21.41	20.85	.614

*Note.* The first column was obtained by averaging $\bar{\Delta E_{\;pm}^{}}$ over the 37 observers for each Smartie sweet type. The second column was similarly obtained for $\bar{\Delta E_{im}^{}}$ . All color differences were calculated in CIE*Lab* following Equations (1) and (2). The third column shows the significance of the values in the first two columns according to paired-sample *t*-tests. The number of observations was *n* =  37 in all cases (see variability of the differences in Figure A4).

**Table 3.** Mean of all pairwise differences $\bar{\Delta E}$ (as defined by Equations (1) and (2), averaged over the five repetitions), with their related p-values. Laboratory measures and outside measures are treated separately*.*

Smartie sweet type	Perception vs. Measured $\left(\bar{\Delta E_{\;pm}^{}}\right)$	Industry vs. Measured $\left(\bar{\Delta E_{im}^{}}\right)$	*p*
Laboratory measures only			
Blue	13.55	17.06	.016
Green	15.15	12.71	.003
Red	23.25	18.76	.001
Yellow	16.60	13.58	.003
Outside measures only			
Blue	22.66	28.15	<.001
Green	25.75	30.15	.036
Red	28.19	33.42	.016
Yellow	25.77	27.99	.234

*Note.* The top part of the table shows results considering only color matchings made inside the laboratory. The bottom part considered only matchings made outside of the laboratory. As before, the first column was obtained by averaging $\bar{\Delta E_{\;p-m}^{}}$ over the 39 observers for each Smartie sweet type. The second column was similarly obtained for $\bar{\Delta E_{i-m}^{}}$ . All color differences were calculated in CIE*Lab* following Equations (1) and (2). The third column shows the significance of the values in the first two columns according to a paired-sample *t*-test. The number of observations was *n * =  39 in all cases. For information on the variability of the differences, see Figure A4.

In the two-dimensional case, the Minimum energy test and the Hotelling T-squared test revealed that both chromaticity distributions, perception-based and industry-based, differed significantly from the measurements (see Table 1 and Gaussian fits in Figure 6). The only exception was the pair perception vs. measured for the yellow Smartie sweet types. In other words, none of the gamma correction methods produced similar distributions of chromaticity values as that measured by the chromameter.

When we looked at the aggregation of individual distances in CIE*Lab* (see Table 2 and Figure A4), the average of *DE* values was closer to zero (i.e., closer to chromameter measurements) for blue Smartie sweet type using the perceptual gamma correction. Similarly, the *t*-test indicated that the difference between the two conversion methods was only significant for the blue Smartie sweet type (*p *< .001).

### Effect of the Different Variability Sources

We explored the changes in color estimation due to gamma correction and how they compare with other variability sources. Since all measurements and matchings were repeated five times in similar conditions, we calculated a mean value (*m*), a standard deviation (s) and a standard error (e), where ( $e=\sigma /\sqrt{n}$ , *n* = 5). Variability was expressed in terms of the relative error (d) by simply dividing e in the mean value of the measurement considered (d = e /*m*), which allowed us to compare values in different numerical scales. Table 4 shows the relative error estimates for the most important variability sources in our study.

**Table 4.** Relative error estimates for various variability sources (chromameter, Smarties factory colors, observers RGB choice, color conversions and both types of ΔE)*.*

Smartie	Chromameter	Smartie shape	Smartie factory color	CIE *xyY* to CIE*Lab* conv.	Observer RGB choice	ΔE_pm_ var	ΔE_im_ var
type	CIE *xyY*	CIE *xyY*	CIE *xyY*	CIE*Lab*	RGB	CIE*Lab*	CIE*Lab*
Blue	0.001	0.010	0.005	0.004	0.062	0.076	0.058
Green	0.001	0.014	0.009	0.001	0.142	0.148	0.134
Red	0.002	0.011	0.015	0.003	0.125	0.088	0.083
Yellow	0.001	0.009	0.008	0.003	0.115	0.109	0.101
All	0.001	0.011	0.009	0.003	0.111	0.105	0.094

*Note*. To calculate the variability of the responses, we used the relative error, which is defined as standard error divided by the average data value.

We considered five main sources of variability, as follows:

1. Chromameter. Refers to the small variations in the results of the same measurement, usually within the tolerance of the instrument. The chromameter variability was estimated by measuring the same screen patch five times in the instrument's native color space (CIE *xyY*).
2. Smartie shape. Given that Smarties are smooth ellipsoidal objects, they produce color gradients and specular reflections that may introduce noise in the color matching task. The variability coming from the Smartie ellipsoidal shape and its specular reflections was estimated by measuring five times the same Smartie sweet type with the chromameter, maintaining approximately the same viewing angle and distance.
3. Smartie factory colors. We tested the variability introduced by small manufacturing differences of Smarties of the same type. The variability of the Smarties’ factory colors was estimated by measuring five different Smarties of the same type with the chromameter.
4. RGB to CIE*Lab* color conversion. We estimated the effects of color conversion (see Figure 3) on the variability already present in the measuring instrument. The variability of the RGB-to-CIE*Lab* conversion was estimated by applying the equations shown in Figure 3 to the five chromameter measurements of a similar patch and averaged for all observers.
5. Observer RGB choice. Observers repeated each color matching five times in similar conditions, and we estimated the variability of their selected RGB values. The variability of the observer's color-picker RGB choices was calculated from the five color matching repetitions under the same conditions and averaged for all observers. The relative error corresponding to ΔE_pm_ and ΔE_im_ was calculated considering the five repetitions and later averaged for all observers.

### Effect of Particularly Noisy Measures

We tested whether these results were produced by unreliable measures or a particularly noisy testing condition. To do so, we recalculated ΔE_pm_ and ΔE_im_ after removing the outliers in each of the five matching repetitions. For each group of repetitions, outliers were defined as measures whose absolute deviation from the median was larger or equal to three times the median absolute deviation (MAD). This definition led to the removal of 120 measures (out of 780). We also wanted to assess the magnitude of outlier effects both within and outside the laboratory setting. Table A2 presents identical results to the last two columns of Table 4, with the exclusion of these outlier measures. Additionally, we categorized the results into measures conducted within the laboratory and those conducted outside the laboratory.

## Discussion

Anyone who wants to study color scientifically faces the challenge of describing and defining this sensory experience. To know what participants actually see and not only what monitors display, one must transform the device-dependent color systems (e.g., RGB) to device-independent color systems (e.g., CIE*Lab*, CIE *xyY*, CIE *LCh*), a process that includes a *gamma correction*, often performed using light-sensitive devices, and a matrix transformation. If the environmental conditions remain the same, the monitor parameters must be measured only once, simplifying the complexity of the study requirements. As a consequence, color studies are usually run under highly controlled laboratory conditions, implying that participants have to come to a particular location, limiting the number and diversity of the populations studied (Elliot et al., 2007; Jonauskaite, Parraga, et al., 2020; Maule & Franklin, 2015; Thorstenson et al., 2022; Wilms & Oberfeld, 2018; Witzel et al., 2019). Here, we wanted to know whether we could be more flexible and replace the tedious and expensive color measurement with a perceptual task like the one used by Colombo and Derrington (2001). A further alternative is the use of the industry-standard gamma of 2.2. Although performed in some color studies, such a transformation relies on several assumptions (Fdhal et al., 2009), which are only sometimes fulfilled in online studies.

To compare the two gamma correction methods (i.e., perception-based task and industry-standard), we simulated an online study (a set of color selections done by untrained participants in diverse settings). To this end, using a computerized color picker program (Jonauskaite et al., 2016), our participants matched the colors of real objects (blue, green, red, and yellow Smartie sweet types) to colors displayed on a computer monitor. Some of them completed the study in the laboratory. In contrast, others did it outside the laboratory (i.e., indoors and outdoors at a local university) using different computer monitors to diversify the testing situations.

We hypothesized that the perception-based gamma estimation task would be worth the effort if the difference between its results and the chromameter results were smaller than the difference between the results obtained via the industry-standard and the chromameter results. Moreover, if the industry-standard gamma value produced color values close to the chromameter-measured color values, then the industry-standard should be preferred because it is a much quicker, simpler, and cheaper method to implement. The results from Table 1 and Figure 6 confirm that the centroids and the raw distributions obtained by the perception-based and the industry-standard CIE*Lab* conversions differed from those obtained by the chromameter. This large difference reached almost 24 CIE*Lab* units for the red Smartie sweet types, which is more than ten times the established JND in the chromaticity (*a,b*) plane.^4^

**Figure A5.**
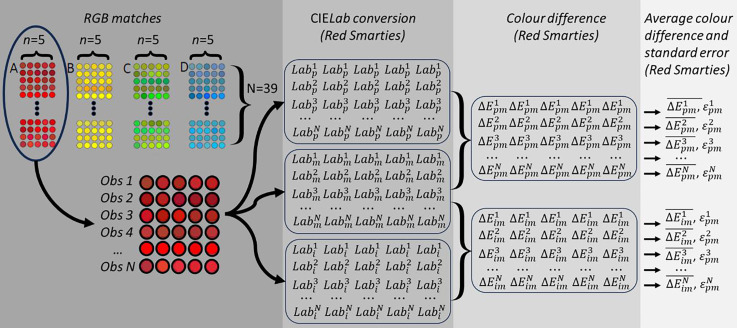
Schematics of how variability was calculated in terms of the relative error. The first panel from the left shows the schematics of the raw RGB matches obtained from the color picker. There were 39 observers (N = 39) and five repetitions of matchings with the same Smartie sweet type (*n* = 5). The second panel from the left represents the resulting CIE*Lab* values obtained from converting these RGB matches using the two conversion methods (sub-index *p* for perception-based and subindex* i* for industry-based) and the direct screen measurement (sub-index *m*). The third panel represents the color differences obtained from these values using Equations (1) and (2). Each row corresponds to one observer, and each column to one repetition. In the last panel, we average these repetitions ( $\bar{\Delta E_{\;pm}^{}}$ , $\bar{\Delta E_{im}^{}}$ ) and obtain the corresponding standard errors $\varepsilon_{\;pm}^{}$ and $\varepsilon_{im}^{}$ . These were later divided to calculate the relative errors.

To analyze the chromatic difference (whole of CIE*Lab*), we calculated Δ*E_pm_* and Δ*E_im_* as described in Figure A5. We averaged these results across subjects in Table 2, with values reaching 26 CIE*Lab* for units for the red Smartie sweet types. The third column of Table 2 shows that the differences between both the perception-based conversion and the industry-based conversion were not statistically significant (*p* > .05), except for the blue Smartie sweet types (*p* < .001). A closer look, disaggregating laboratory and outside measures paint a different picture. Table 3 shows that color differences obtained in the laboratory were systematically smaller than outside. Indeed, the largest difference for measurements obtained outside was 33.42 CIE*Lab* units (again for the red Smartie sweet types). Table 3 also shows that if we discriminate between laboratory and outside measures, the differences between both conversion types become significant (*p* < .05 in all cases, except for yellow Smartie sweet types measured outside the laboratory). The same results show that the industry-standard gamma conversions generally obtained values closer to the measurements than the perception-based conversions inside the laboratory. Still, the opposite occurred outside (i.e., the perception-based conversions were closer to the chromameter measurements than the industry-standard gamma conversions). The fact that the supremacy of one conversion method over the other reverses depending on the prevailing environmental conditions suggests that other factors may impact the outcome.

### Factors Potentially Influencing Our Results

The previous results prompted us to investigate factors beyond gamma correction that might have influenced our results. Table 4 displays the average relative errors associated with various sources of variability that may affect our calculations. Notably, the first four columns exhibit values one or two orders of magnitude smaller than those in the last three columns. For example, the variability in chromameter measurements of identical screen patches (Chromameter column) ranges between 0.1% and 0.2%, well within the typical tolerance of most instruments (0.5%). The variation attributed to specular reflections and chromatic gradients due to the ellipsoidal shape of the Smartie sweet types was also minimal (between 0.9% and 0.14%). The variability due to individual factor differences between Smartie sweet types was also in the same range (between 0.5% and 0.15%). Indeed, the largest source of variability comes from the observers’ color picker choices (Observer RGB choices column), with values of the same order of magnitude (between 6% and 14%) as those of the color differences, ΔE_pm_ and ΔE_im_ (between 7% and 15%).

We also looked at the RGB variability between color matches obtained inside and outside the laboratory (see Appendix Table A1) and found them to be very similar (laboratory matches between 6% and 15% and outside matches between 6% and 13%). Indeed, we expected subjects inside the laboratory to have less variability in their RGB matches, but that was not the case.

Since the relative error tends to add up in multiplications (Kirchner, 2001), we must assume that the variability observed in subsequent calculations, such as color differences, is a cumulative result of the variabilities encountered in preceding stages (chromameter, Smartie sweet shape, observer RGB matching, etc.). This implies that the relative error introduced by the instrument, the Smartie sweet types, and the color conversions is likely to be negligible when compared to the error due to observer variability in the color-matching task. For instance, some participants might have misunderstood the instructions, lacked task engagement, or found the task too difficult. These performance errors would result in less accurate gamma estimations, enhancing differences between the perception-based gamma estimation task and the chromameter measurements. We tested whether these results were due to particularly bad measures. Removing outliers produced an improvement in the color difference variability but did not reverse the overall trend of the results. Table A2 shows a slight improvement in all measures (5%–13%), laboratory measures (3.5%–14%), and outside measures (7%–12%). Interestingly, eliminating outliers led to a more pronounced improvement in the variability of outside measurements compared to laboratory measurements.

Further variance was also possible. Regarding the industry-standard gamma, the value of 2.2 applies to most monitors worldwide (Poynton & Johnson, 2004). However, Apple is presetting their Macintosh monitors to an industry-standard gamma of 1.8 (Poynton, 1996). This difference could explain some deviations from the chromameter measurements. Also, the standard gamma does not hold in the conditions that significantly deviate from the default ones. Regarding the chromameter measurements, they likely varied because of the inherent precision of the instrument and changes in the measurement conditions—lighting, reflections, temperature, monitor angles, experimenter handling the device, etc. For example, for the most accurate results, the chromameter must be pointed perpendicularly to the target color at an eye distance of a participant, and the color sample must be sufficiently large. Putting the chromameter in a stable position, such as on a tripod, would further improve these measurements (Parraga et al., 2014). Studies with different experimental designs are necessary to evaluate how these different sources of variability might influence the accuracy of the gamma estimation methods.

### Conclusions and Future Directions

Taken together, our results show that neither the perception-based task nor the industry-standard resulted in color values identical to the chromameter measurements. Thus, if the chromameter acts as the gold standard, it should be favored whenever possible. When the chromameter is unavailable, like in online studies, our results demonstrated that none of the alternative methods (i.e., the perception-based and the industry-standard) was superior to the other. The differences between the two methods were small in magnitude, dependent on color, and, crucially, extremely minor compared to the uncertainty introduced by human observers. Thus, to choose between the two alternatives (i.e., perception-based or industry-standard), it is necessary to initially assess the variability among untrained observers in the given task, followed by an evaluation of whether standard viewing conditions can be assumed or not. When observers are trained, and the task is well defined, researchers can assume relatively standard viewing conditions (e.g., right-angle viewing, stable illumination, etc.). Still, when the colorimetric properties of the screens are unknown (e.g., miscellaneous LCD monitors, tablets, or cell phones), the perception-based task should be more advantageous. In all other cases, the simpler industry estimation method seems sufficient.

For now, we suggest that researchers who are unable to perform chromameter measurements run experimental color studies under maximally controlled viewing conditions. These conditions should match the default conditions assumed for the industry-standard gamma (Anderson et al., 1996). These default conditions refer to a testing environment out of direct sunlight, usually the typical viewing environment of an office, which is illuminated with daylight or D-65 artificial light. Then, it would be important to turn off any filters affecting color display (e.g., Flux, Night mode, etc.) and to set monitors to a comfortable but relatively high brightness. Complying with such requirements should enhance the likelihood that the sRGB color standard is met, making color conversions with the industry-standard gamma more accurate.

To learn how broadly our conclusions hold, future studies should consider diverse groups of participants (not only Swiss university students) by including those who are less used to computer tasks (e.g., children, elderly) and consider inter-individual factors like participants’ visual sensitivities. Future studies should also go beyond the four color categories (i.e., red, yellow, green, blue) and consider colors produced more frequently in psychological color studies (see the diversity of possible colors here, Jonauskaite, Althaus et al., 2019; Jonauskaite, Dael et al., 2019; Jonauskaite et al., 2016; Prado-León et al., 2014; Zhou et al., 2022). All in all, the current study should help future researchers to choose the best monitor calibration method within their realm of possibilities and hopefully make psychological color studies less daunting to run.

## Figures and Tables

**Figure A3.**
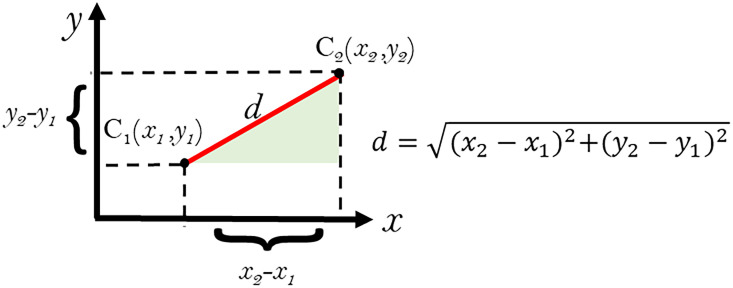
Calculation of a Euclidean distance (d) between two points (i.e., color selections) in the CIE *xy* color space.

Color differences were averaged over similar repetitions (*n* = 5) before computing the boxplots. Panels A, B, C and D correspond to blue, green, red, and yellow Smartie sweet types. Boxplots show median value, 25th and 75th percentiles (lower and upper hinges), the lowest measured values within Q1 (first quantile), and 1.5 × Q1 (lower whisker) and the highest observed value within Q3 (third quantile) and 1.5 × Q3 (upper whisker).

## Appendix

### Creation of Half-Tone Patterns and Square Patches for the Perception-Based Gamma Estimation Task (Phase 1)

We started with eight half-tone patterns of different luminance levels, each consisting in 3 × 3 pixel arrangements (see an example in Figure A1). For each half-tone pattern, we changed the number of ON pixels, and did so for each color channel (RGB), separately. The ON pixels had a maximum value of 255 while the OFF pixels were set to zero. Thus, for the Red channel, the ON pixels had a value of RGB 255, 0, 0, appearing in bright red. For the Green channel, the ON pixels had a value of RGB 0, 255, 0, appearing in bright green, and for the Blue channel, the ON pixels had a value of RGB 0, 0, 255, appearing in bright blue. In all cases, the OFF pixels had a value of RGB 0, 0, 0, appearing in black.

The half-tone 3 × 3 patterns emitted an increasing fraction of nine of the maximum possible luminance—1/9 (one pixel ON), 2/9 (two pixels ON), 1/3 (three pixels ON), 4/9 (four pixels ON), 5/9 (five pixels ON), 2/3 (six pixels ON), 7/9 (seven pixels ON), and 8/9 (eight pixels ON) (see Figure A1). For example, if a monitor emitted 90 Cd/m^2^ at its maximal luminance, the half-tone pattern of 1/9 would emit 10 Cd/m^2^ (i.e., 90/9 Cd/m^2^). Thus, there were eight half-tone patterns for each color channel (RGB), resulting in 24 half-tone patterns in total. In other versions of the same method, we also used four repetitions and 16 repetitions per color. We opted for eight repetitions to balance between the length of the task and the reliability of estimations.

We then spatially multiplied the respective half-tone patterns 69 times so to form larger squared patches consisting of 207 × 207 pixels. Doing so for all color channels meant that we had 24 square patches in total (8 fractions × 3 colors). At the center of each patch, we placed a small circle and a concentric ring, made of pixels of the same chromaticity either set to maximum or minimum brightness (see Phase 1 in Figure 1). Therefore, these circles and rings appeared either darker or brighter than the background made of the half-tone patterns (see also, Parraga et al., 2014).

### Relative Error as a Measure of Variability

To estimate the variability for the different sources of noise we took advantage of the repeated measures that observers made in every single condition. We calculated variability as the quotient between the standard error and the average of these five repetitions, which allowed us to compare all different variability sources in Table 4. For example, in the case of the RGB values, we collected five matchings for every Smartie sweet type and observer and obtained the averages $\bar{R},\bar{G},\bar{B}$ and the standard errors $\varepsilon^{R},\varepsilon^{G},\varepsilon^{B}$ over these five repetitions. We then calculated variability by averaging the relative errors $avg\left(\frac{\varepsilon^{R}}{\bar{R\;}},\frac{\varepsilon^{G}}{\bar{G\;}},\frac{\varepsilon^{B}}{\bar{B\;}}\right)$ as a single value for each observer and Smartie type. Since some observers made their measures outside, and others in the laboratory, were collected the averages for all, outside and in the lab in Table A1. A similar procedure was used to calculate the variability of CIE *xyL-*based measures in Table 4.

**Table A1.** Variability of the raw RGB matchings obtained by the color picker, discriminated by location (all, laboratory, outside) and Smartie sweet type (blue, green, red, yellow)*.*

Smartie sweet type	Observer RGB choice		
	All	Laboratory	Outside
Blue	0.062	0.062	0.061
Green	0.142	0.158	0.125
Red	0.125	0.113	0.136
Yellow	0.115	0.117	0.113

*Note*. The case of average color differences ( $\bar{\Delta E_{\;pm}^{}}$ and $\bar{\Delta E_{im}^{}}$ ) was slightly more complicated. Figure A5 shows the schematics of how variability was calculated in this case. The left part of the figure illustrates the different color matchings made in RGB using the color picker (*n* = 5 repetitions and *N* = 39 observers). For the sake of simplicity, we focused on red Smartie sweet types only. These were converted to CIE*Lab* as described above using the two conversion methods (perception-based and industry-based gamma correction) which are identified by sub-indices *p* and *i* respectively. Sub index *m* (for *measured*) refers to the CIE*Lab* values obtained by pointing the chromameter to the resulting screen patch and converting these CIE*xyL* measures into CIE*Lab* (no gamma correction is needed here). The next step is to obtain the color differences *(*Δ*E_pm_ and* Δ*E_im_*) between the colors produced by each of the conversions and the corresponding measurement. Finally, the results of the five repetitions were averaged and the values of $\bar{\Delta E_{\;pm}^{}}$ , $\bar{\Delta E_{im}^{}}$ , $\varepsilon_{\;pm}^{}$ and $\varepsilon_{im}^{}$ were obtained for each observer. From these, we calculated the corresponding relative errors as the quotients $\frac{\varepsilon_{\;pm}^{}}{\bar{\Delta E_{\;pm}^{}\;}}$ and $\frac{\varepsilon_{im}^{}}{\bar{\Delta E_{im}^{}\;}}$ . These were averaged for all observers and presented in Table 4. Averages including laboratory-only and outside-only measures were presented in Table 3.

### High Variability Measures Removal

Although this research intended to emulate the conditions and the variability of a typical “in the wild” experiment, we repeated the analysis after eliminating some of the most problematic measures. The rows of five dots in Figure 4 show representations of the RGB values selected by each observer when asked to match a Smartie sweet type five times. In theory, the colors of these dots should be the same, but clearly, they were not. We converted these colors to CIE*Lab* and eliminated measures whose absolute Δ*E* deviation from the five-values median was larger or equal to three times the median absolute deviation (MAD). Using these criteria, we eliminated 120 out of 780 measures. Table A2 shows the variability of Δ*E_pm_* and Δ*E_im_* after removing those values.

**Table A2.** Relative error estimates for both types of ΔE, after removing outliers*.*

Smartie sweet type	*DE_pm_* var	*DE_im_* var
All conditions		
Blue	0.064	0.050
Green	0.133	0.113
Red	0.082	0.068
Yellow	0.086	0.078
Laboratory		
Blue	0.057	0.035
Green	0.146	0.131
Red	0.062	0.066
Yellow	0.078	0.092
Outside		
Blue	0.070	0.067
Green	0.120	0.093
Red	0.103	0.071
Yellow	0.095	0.064

*Note*. Outliers were removed from each of the five-repetitions as follows: we calculated the median and the median absolute deviation (MAD) and removed values whose distance from the median was equal or larger than three MADs.

## Notes

1. We chose the CIE*Lab* space because it was designed to achieve perceptual uniformity, where a numerical difference corresponds to a comparable perceived change in color. Also, its units were originally designed to correspond to the just noticeable difference or JND, but subsequent amendments recommend using approximately 2.3 CIE*Lab* units as a practical JND measure (Gaurav, 2003). It is important to note that although the space is not entirely perceptually uniform, it remains the standard for discerning subtle color variations.

2. The estimation task is accessible here: https://www2.unil.ch/onlinepsylab/ColourPicker/html/colorpicker.html (under the tab “Calibration”), and the full code is available here: https://osf.io/hs7p2/.

3. https://www5.konicaminolta.eu/fr/instruments-de-mesure/produits/lumiere-et-ecrans/produits-discontinues/cs-100a.html.

4. The distributions of raw values in Figure 5 show a gamut contraction (closer points) between the CIE*Lab* values calculated from the chromameter screen measures (panels A and D) and the same values converted using Equations (1) and (2) (other panels). This is expected since the matrix and equations in Figure 3 were optimized for a standard sRGB-to-CIE*Lab* color transformation, and there is no guarantee that the screens used in our experiments fully support the sRGB specifications. Nevertheless, we chose to operate in CIE*Lab* because of its uniformity and the link between its units and perceptual JND.
